# Immunomodulatory mAbs as Tools to Investigate on Cis-Interaction of PD-1/PD-L1 on Tumor Cells and to Set Up Methods for Early Screening of Safe and Potent Combinatorial Treatments

**DOI:** 10.3390/cancers13122858

**Published:** 2021-06-08

**Authors:** Cinzia Vetrei, Margherita Passariello, Guendalina Froechlich, Rosa Rapuano Lembo, Nicola Zambrano, Claudia De Lorenzo

**Affiliations:** 1 Department of Molecular Medicine and Medical Biotechnology, University of Naples “Federico II”, 80131 Naples, Italy; cinzia.vetrei@unina.it (C.V.); margherita.passariello@unina.it (M.P.); r.rapuanolembo@studenti.unina.it (R.R.L.); zambrano@unina.it (N.Z.); 2 Ceinge—Biotecnologie Avanzate s.c. a.r.l., via Gaetano Salvatore 486, 80145 Naples, Italy; guendalina.froechlich@unimi.it; 3 European School of Molecular Medicine, University of Milan, 20122 Milan, Italy

**Keywords:** immune checkpoints, immunomodulatory mAbs, cancer immunotherapy, cardiotoxicity, irAEs

## Abstract

**Simple Summary:**

A novel challenge in cancer immunotherapy is the identification of the most potent combinations of immunomodulatory mAbs that are capable of maximizing therapeutic benefits while minimizing irAEs. We set up an in vitro system to quickly predict the efficacy and eventual cardiotoxic side effects of combinatorial treatments, thus allowing for the early screening of most potent and safe combinatorial therapeutic regimens for both validated and emerging immunomodulatory mAbs against different immune checkpoints (ICs). Furthermore, we provide for the first time evidence on cis-interactions of ICs in tumor cells.

**Abstract:**

Antibodies targeting Immune Checkpoints (IC) on tumor infiltrating lymphocytes improve immune responses against cancer. Recently, the expression of some ICs has also been reported on cancer cells. We used the clinically validated Ipilimumab and Nivolumab and other novel human antibodies targeting Cytotoxic T- lymphocyte-antigen 4 (CTLA-4), Programmed Death receptor-1 (PD-1) and Programmed Death Ligand 1 (PD-L1) to shed light on the functions of these ICs in cancer cells. We show here for the first time that all these antagonistic mAbs are able to reduce Erk phosphorylation and, unexpectedly, to induce a significant increase of ICs expression on tumor cells, involving a hyperphosphorylation of NF-kB. On the contrary, agonistic PD-L1 and PD-1 recombinant proteins showed opposite effects by leading to a significant reduction of PD-1 and PD-L1, thus also suggesting the existence of a crosstalk in tumor cells between multiple ICs. Since the immunomodulatory mAbs show their higher anti-tumor efficacy by activating lymphocytes against cancer cells, we also investigated whether it was possible to identify the most efficient combinations of immunomodulatory mAbs for achieving potent anti-tumor efficacy associated with the lowest adverse side effects by setting up novel simple and predictive in vitro models based on co-cultures of tumor cells or human fetal cardiomyocytes with lymphocytes. We demonstrate here that novel combinations of immunomodulatory mAbs with more potent anti-cancer activity than Ipilimumab and Nivolumab combination can be identified with no or lower cardiotoxic side effects. Thus, we propose these co-cultures-based assays as useful tools to test also other combinatorial treatments of emerging immunomodulatory mAbs against different ICs for the early screening of most potent and safe combinatorial therapeutic regimens.

## 1. Introduction

Cancer Immunotherapy, aimed at boosting natural body’s defense to fight cancer, is based on antibodies either directed against tumor associated antigens overexpressed on cancer cells, to inhibit their growth, or Immune Checkpoints (ICs), in order to activate T-cells against cancer. Immune-checkpoint inhibitory antibodies approved by the Food and Drug Administration (FDA), such as Nivolumab and Ipilimumab [1,2], have represented the most important clinical success in cancer therapy over the last decade.

The human mAb, Nivolumab, recognizes the Programmed Death receptor-1 (PD-1), that is expressed on T-cells where it exerts a crucial role in immune suppression [3,4] upon its binding to its natural ligand, Programmed Death Ligand 1 (PD-L1), which is expressed on Antigen Presenting Cells (APC) and on cancer cells [5].

The role of PD-L1 on tumor cells is not only involved in tumor escape from immune surveillance, but also in tumor cell proliferation and survival through the activation of Mitogen Activated Protein Kinase (MAPK) pathway [6]. PD-L1 expression on cancer cells can be directly modulated by nuclear factor kappa-light-chain-enhancer of activated B cells (NF-kB), a master transcription factor of inflammation. NF-kB not only binds to PD-L1 promoter by inducing its transcription [7], but it can also improve its post-translational stabilization by inducing the expression of proteins such as COP9 signalosome subunit 5 (CSN5) [8].

The Cytotoxic T- lymphocyte-antigen 4 (CTLA-4), is another IC targeted by the inhibitory human antibody Ipilimumab, of particular success in clinical use for the treatment of metastatic melanoma [9,10] and for other solid tumors, such as non-small-cell lung cancer (NSCLC), and renal cell and prostate carcinomas [11]. CTLA-4 competes with T-cell associated CD28 receptor for the binding with B7-1/B7-2 ligands [12], expressed on APC.

Moreover, CTLA-4 is expressed on regulatory T cells (T reg) and Natural Killer (NK) cells, even though its role in the latter population has not been yet fully elucidated [13,14]. In recent studies, CTLA-4 expression has been reported also on non-immune cells such as tumor cells, where it has been supposed to play a role in survival, by promoting proliferation or inducing apoptosis [15].

Ipilimumab and Nivolumab have shown efficacy and have been approved for the treatment of several tumors [16]; however their average response rate of 10–15% [17] and 31–44%, respectively [18] is still limited. Thus, combinatorial treatments to achieve higher therapeutic index with respect to monotherapies have been tested by several clinical trials demonstrating several benefits in terms of overall survival [19] but also increased side immune-related adverse events (irAEs) [20,21,22,23,24].

A novel challenge in cancer immunotherapy is the identification of the most potent combinations of mAbs which are capable of maximizing therapeutic benefits while minimizing irAEs. Although immunomodulatory mAbs, such as the anti-PD-1 mAbs, are frequently associated with a wide spectrum of immune-related adverse events, cardiac toxicity has not been properly studied.

The combined treatment of anti PD-1 and anti CTLA-4 antibodies showed major side effects as in the reported cases of myocarditis [25], whereas cases of pericarditis and cardiomyopathies have also been observed during treatment with Ipilimumab alone [26]. To provide a further progress in the field, we generated a large repertoire of fully human antibodies specific for 10 different ICs [27,28] through a novel selection strategy of phage libraries on human activated lymphocytes expressing the ICs in their native conformation, which allowed the collection of mAbs against multiple targets in one single panning. Subsequent screening was performed by using purified proteins and by next generation sequencing (NGS). Human IgGs from four of these collections (i.e., PD-1, PD-L1, Lag-3 and CTLA-4), were characterized and found to specifically bind to their targets with high affinity, to efficiently activate T cell proliferation, to induce cytokine secretion and inhibit in vivo tumor growth. Interestingly, the novel isolated mAbs have comparable or even better binding affinity and biological activity than the clinically validated anti-PD-1 mAb Nivolumab [6,27,28].

Herein, in order to explore the possibility to enhance the antitumor effects of the novel immunomodulatory antibodies we tested them in appropriate combinations among them or with the available FDA approved mAbs to assess the most potent and safe combinatorial approach by performing preclinical studies in simple in vitro models set up on co-cultures of tumor or cardiac cells and lymphocytes. Furthermore, we used these mAbs as biotechnological tools to shed light on the role and crosstalk of different ICs in tumor cells, revealing for the first time intriguing interconnections between them in tumors, thus opening new scenarios for cancer therapy.

## 2. Results

### 2.1. Effects of Different Immunomodulatory mAbs on a Panel of Tumor Cells

It has been reported in literature that some Immune Checkpoints, such as PD-1, PD-L1 and CTLA-4, are expressed not only on immune cells but also on many different types of tumors, and that immunomodulatory mAbs specific for these targets can affect tumor cell viability even in the absence of immune cells [6,28,29,30,31]. Here, we investigated the effects of anti-PD-1, anti-PD-L1 and anti-CTLA-4 immunomodulatory mAbs on different tumor cells by choosing in particular breast and lung cell lines, as many ongoing clinical trials are evaluating the efficacy of immunomodulatory mAbs on these tumors [32,33,34].

**Figure 1:**
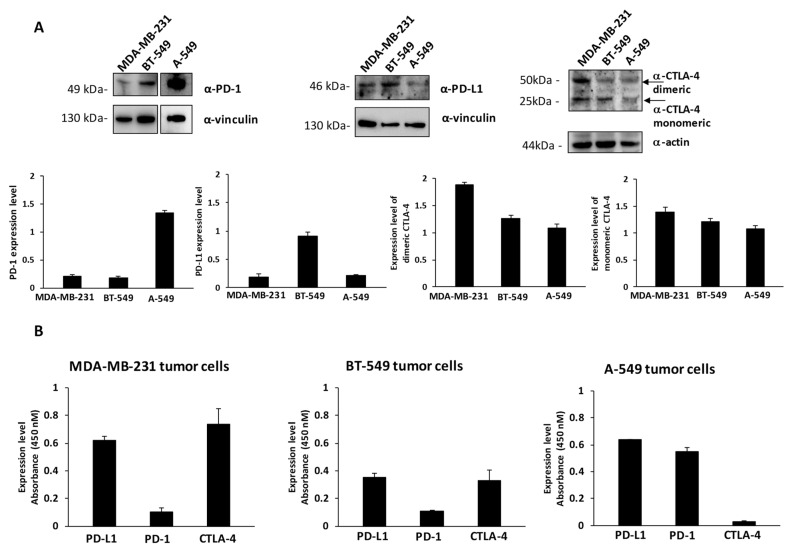
Expression of PD-L1, PD-1 and CTLA-4 ICs on cancer cells. (**A**) Western blotting analyses of extracts from MDA-MB-231, BT-549 and A-549 tumor cells, by using the commercial anti-PD-L1, anti-PD-1 or anti-CTLA-4 mAbs; the arrows indicate the dimeric and monomeric forms of CTLA-4. Full Western blots are included in the Figure S3. The intensity of the bands corresponding to ICs was normalized to actin or vinculin and their ratio is reported in the graphics as protein expression levels. (**B**) Cell ELISA assays were performed on whole cells to measure the cell surface expression of ICs with commercial anti-PD-1, anti-PD-L1 or anti-CTLA-4 mAbs on MDA-MB-231, BT549 or A-549 tumor cells. Binding values were reported as the mean of at least three determinations obtained in three independent experiments. Error bars depicted means ± SD.

To this aim, we first checked the levels of expression of PD-1, PD-L1 and CTLA-4 on MDA-MB-231 and BT-549 breast cancer cells, and A-549 lung cancer cells by cell ELISA to measure the levels of these ICs exposed on the cell surface and by Western blotting to measure the total amounts of the proteins by using the commercial anti-PD-1, anti-PD-L1 and anti-CTLA-4 mAbs at a concentration of 200 nM (see Figure 1A,B). These cell lines express satisfactory levels of the selected Immune checkpoints, in particular the expression levels of PD-L1 are comparable between the three indicated cell lines, whereas the levels of PD-1 are much higher in A-549 cells with respect to the other two cell lines. MDA-MB-231 cells express the highest level of CTLA-4, which is present in both dimeric and monomeric forms, as previously reported [28], whereas A549 seem to express the lowest levels of CTLA-4 on the cell surface (Figure 1B). As negative and positive controls untreated or activated lymphocytes expressing the three ICs on the surface were used [14,27].

Once we confirmed the significant expression of these ICs in two out of three cell lines, we tested the effects of the human monoclonal antibodies, currently in clinical use for cancer treatment, [35] Nivolumab and Ipilimumab (anti-PD-1 and anti-CTLA-4 respectively) and of other human anti-PD-L1 (10_12 and PD-L1_1), anti-PD-1 (PD-1_1) or anti-CTLA-4 (ID1) immunomodulatory mAbs, previously generated in our laboratory by a novel selection strategy on activated human peripheral blood mononuclear cells (hPBMCs) [6,27,28,36]. These novel antibodies, previously characterized for their binding affinity and specificity for the targets and tested in vitro and in vivo for their biological properties, were reported to have a Kd in the low nanomolar range of 0.1–0.4 nM [27]; however, an affinity matured variant (with a lower Kd) of anti-PD-L1 mAb, called 10_12, was also generated by yeast display [36].

Considering the previous reports [6,28] on the effects of anti-PD-L1 and anti-CTLA-4 mAbs on tumor cell growth, we treated the indicated three different PD-1, PD-L1 and CTLA-4–positive tumor cells for 72 h with 100 nM of PD-L1_1, 10_12, PD-1_1 or ID1 mAbs to compare their biological activity with that of the clinically validated Nivolumab and Ipilimumab mAbs.

The dose of 15 μg/mL (~100 nM), used for the treatments, was chosen on the basis of previous analyses performed with immunomodulatory mAbs [6] and considered comparable to that (15 mg/Kg) used for validated immunomodulatory mAbs in preclinical studies and in clinical practice [37].

**Figure 2:**
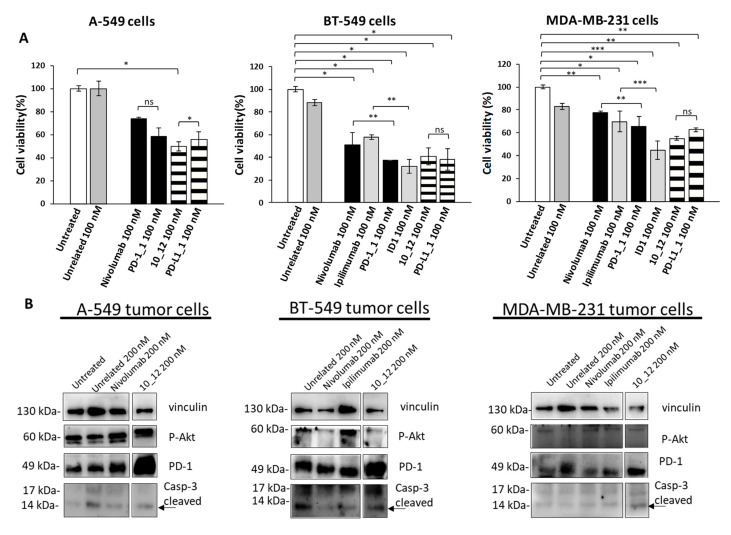

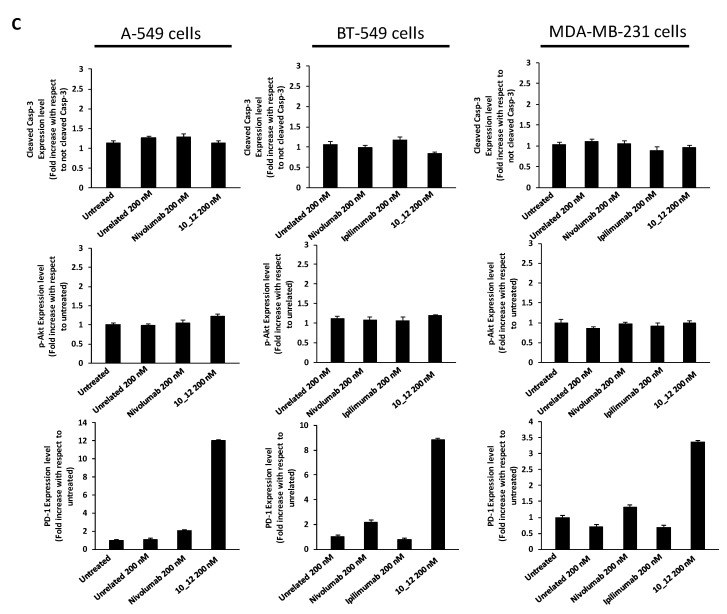
Immunomodulatory mAbs reduce tumor cell viability by affecting intracellular pathways. (**A**) A-549, BT-549 and MDA-MB-231 tumor cells were treated for 72 h with anti-PD-1, anti-PD-L1, anti- CTLA-4 or unrelated (anti-SRB1 or anti-Claudin 1) mAbs at the indicated concentrations. Cell survival is expressed as percentage of viable cells with respect to untreated ones. Cells were counted before and after Trypan Blue exclusion test. The values were reported as the mean of at least three determinations obtained in three independent experiments. Error bars depict means ± SD *** *p* < 0.001; ** *p* < 0.01; * *p* < 0.05; ns, not significant. (**B**) Western blotting analyses of extracts from MDA-MB-231, BT-549 or A-549 tumor cells treated for 72 h as indicated. Images of the whole Western blots are included in Figure S4. The intensity of the bands was normalized to vinculin. (**C**) Densitometry quantification of signals from Western blotting analyses. Protein levels are expressed as fold increase with respect to cells untreated or treated with an unrelated IgG; cleaved Caspase-3 level is expressed as fold increase with respect to uncleaved Caspase-3.

**Figure 3:**
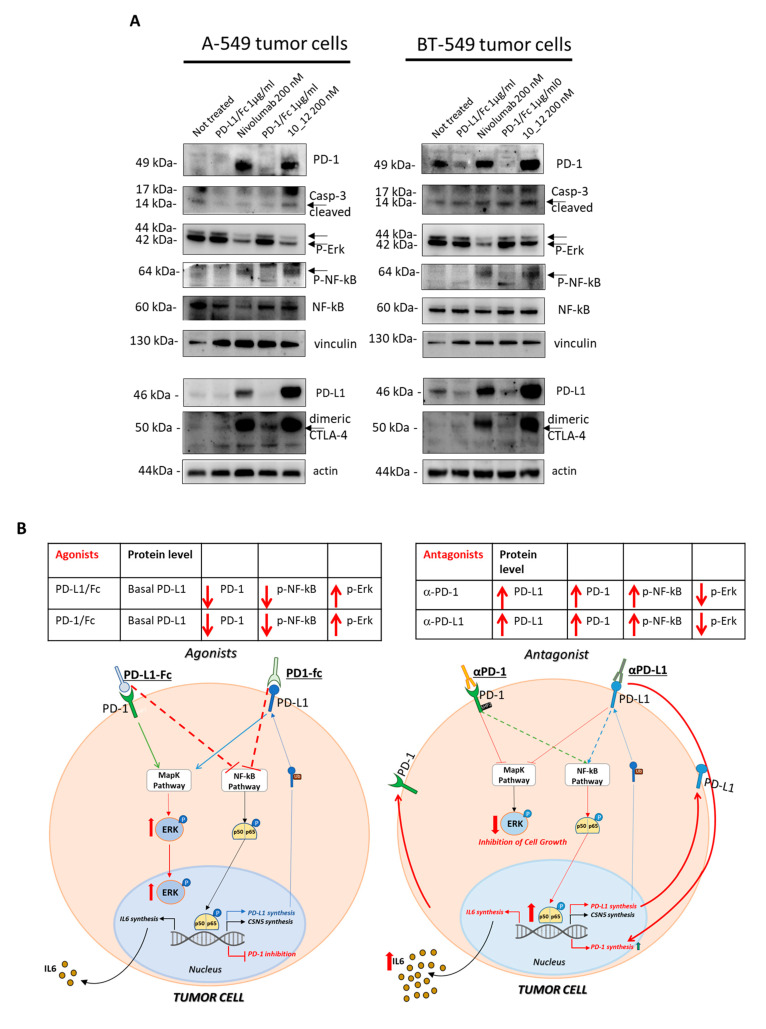
Effects of agonists and antagonists of PD-1 and PD-L1 on tumor cells. (**A**) Western blotting analyses of cell extracts from A-549 or BT-549 tumor cells, treated for 72 h with PD-1/PD-L1 agonists (PD-1/Fc or PD-L1/Fc) or antagonists (Nivolumab or 10_12 mAbs). Images of the whole Western blots are included in Figure S5. The intensity of the bands was normalized to vinculin or actin. (**B**) Model proposed to explain the effects of agonists or antagonists of PD-1 and PD-L1 on tumor cells and on their intracellular pathways.

As reported in Figure 2A, all the tested mAbs show significant effects on tumor cell viability with the strongest effects on BT-549 cells where PD-1_1, ID1, 10_12 and PD-L1_1 reached more than 50% cell growth inhibition with respect to control untreated cells or cells treated with an unrelated control IgG (Figure 2A). To shed light on the anti-tumor effects of these mAbs against three different ICs, in the absence of immune cells, we investigated on the intracellular pathways downstream PD-1, PD-L1 and CTLA-4 by analyzing with suitable commercial antibodies the lysates of tumor cells treated for 72 h with the indicated mAbs at a concentration of 200 nM. This concentration was chosen on the basis of previously reported studies on the effects of immunomodulatory mAbs on intracellular pathways [6,28]. As negative controls we used in parallel assays (by using the same secondary antibody for Western blotting detection) both untreated cells or cells treated with either an unrelated anti-Claudin 1 mAb or an anti-SRB1 mAb. The anti-CTLA-4 mAbs (Ipilimumab and ID1) were not tested on A-549 cells as this cell line does not seem to express sufficient levels of cell surface CTLA-4, as observed by Cell ELISA (Figure 1B). Surprisingly, we found for the first time an enhanced expression of PD-1 receptor when the tumor cells were treated with anti-PD1, anti-PD-L1 or anti-CTLA-4 mAbs (Figure 2B), whereas a decreased phosphorylation of Erk (Figure 3A), that could partially explain the reduction of cell viability, was found after treatment with all of them. No significant effects were observed on Akt phosphorylation or cleavage of caspase-3 (Figure 2B and Figure 3A).

To clarify the role of increased PD-1 expression that we found especially when the cells were treated with the antibodies against PD-1 (Nivolumab) and PD-L1 (10_12), we decided to analyze the cell extracts of tumor cells treated for 72 h with PD-1 or PD-L1 agonists (PD-1/Fc or PD-L1/Fc chimeric proteins) for comparing their effects to those shown by their respective antagonists, Nivolumab or 10_12 mAbs. As shown in Figure 3A, tumor cells treated with the agonists show opposite effects on PD-1 receptor levels by significantly reducing its expression. The antagonists induced again not only an increased expression of PD-1 but also an increased phosphorylation of NF-kB transcription factor, which is reported in the literature [8] to be involved both directly and indirectly in PD-L1 expression on tumor cells (Figure 3A). These findings led us to formulate the hypothesis that in tumor cells a crosstalk between PD-1 receptor and its ligand PD-L1 could occur to support tumor cell survival and, when the PD-1/PD-L1 cis-interaction is blocked, a compensatory increased expression of both the proteins occurs. Indeed, when the PD-L1 or PD-1 induced signaling is affected, as in the case of treatment with antagonistic mAbs, the tumor cells seem to perceive this ligand/receptor unavailability and respond by activating NF-kB hyperphosphorylation, provoking its translocation into the nucleus and likely PD-1 and PD-L1 or CSN5 transcription (see Figure 3B). It could be assumed that NF-kB activity coordinates the expression of both these two ICs, PD-L1 and its receptor PD-1. To test the involvement of mammalian target of rapamycin (mTOR) kinase protein, we also checked the effects of PD-1/PD-L1 agonists and antagonists on its phosphorylation and level of expression, but no significant effects were observed. Unexpectedly, in parallel to the increased expression of PD-1 receptor and its ligand, we also found a significant increase of CTLA-4 expression on the indicated tumor cells after treatment with the antagonists Nivolumab and 10_12, whereas again an opposite effect (decrease in the expression of CTLA-4) was observed under treatments with PD-1/PD-L1 agonists (see Figure 3B). These data suggest the additional involvement of CTLA-4 receptor in the crosstalk existing between PD-1 and PD-L1 ICs, like a common thread of coordination to respond to the effects of the antagonistic or agonistic compounds of PD-1/PD-L1 axis also in tumor cells.

### 2.2. Evaluation of Cytotoxic Effects of Immunomodulatory mAbs on Co-Cultures of hPBMCs and Tumor Cells

**Figure 4:**
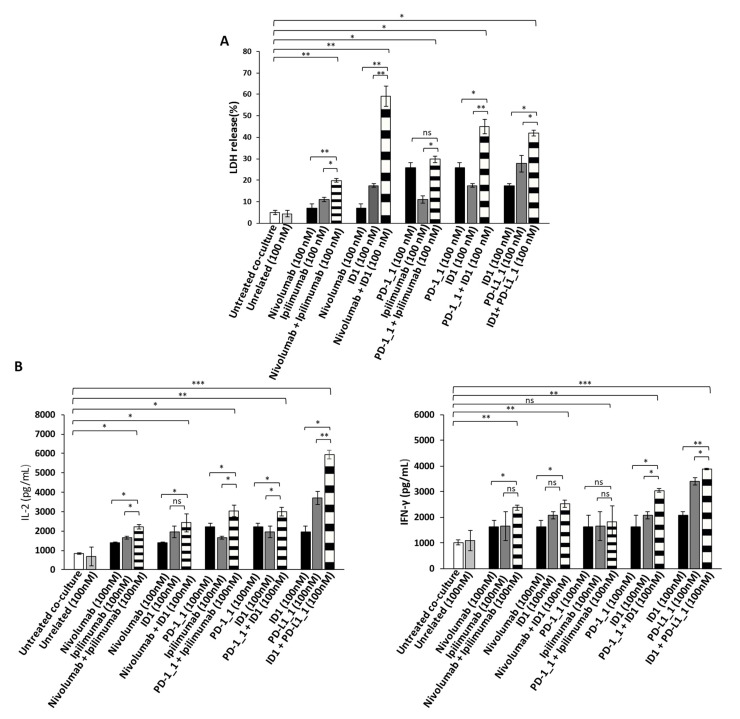
Anti-tumor effects of Immunomodulatory mAbs on BT-549 tumor cells co-cultured with hPBMCs. (**A**) Cell lysis was measured by evaluating the release of Lactate dehydrogenase (LDH) in the supernatants by BT-549 tumor cells co-cultured with hPBMCs for 48 h in the absence or presence of immunomodulatory mAbs, used alone (black and dark grey bars) or in combination (striped bars), at the indicated concentrations. (**B**) The levels of secreted IL-2 and IFN-γ were measured by ELISA on supernatants of co-cultures treated, as indicated. Cytokines are expressed as pg/mL. The values were reported as the mean of at least three determinations obtained in three independent experiments. Error bars depict means ± SD *** *p* < 0.001; ** *p* < 0.01; * *p* < 0.05; ns, not significant.

**Figure 5:**
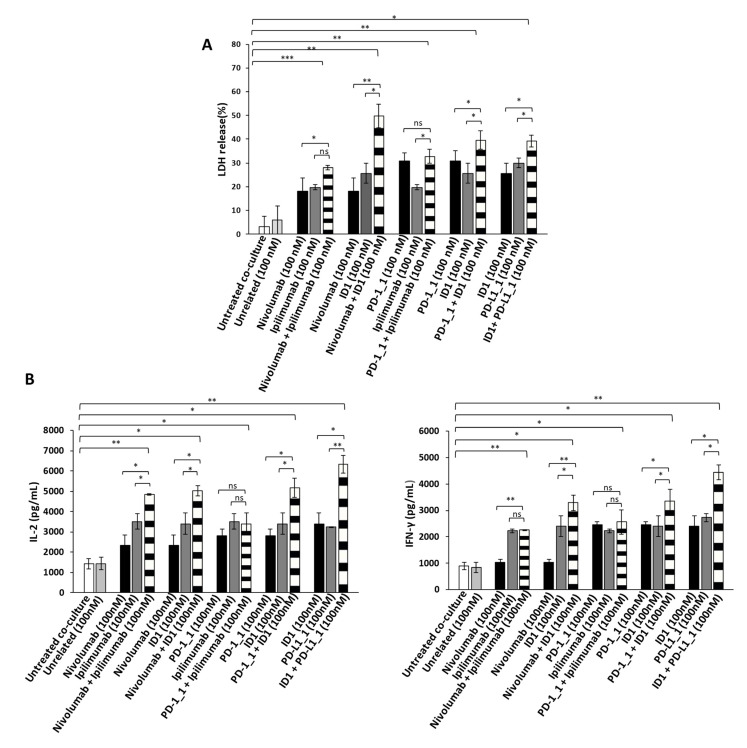
Anti-tumor effects of Immunomodulatory mAbs on MDA-MB-231 tumor cells co-cultured with hPBMCs. (**A**) Evaluation of tumor cell lysis by detecting LDH release from MDA-MB-231 tumor cells co-cultured with hPBMCs, for 48 h in the absence or presence of immunomodulatory mAbs, used alone (black and dark grey bars) or in combination (striped bars), at the indicated concentrations. (**B**) Activation of lymphocytes by anti-PD-1, anti-PD-L1 or anti-CTLA-4 mAbs was analyzed by evaluating the secretion of IL-2 and IFN-γ in the supernatant of the treated co-cultures. Cytokines are expressed as pg/mL. The values were reported as the mean of at least three determinations obtained in three independent experiments. Error bars depict means ± SD *** *p* < 0.001; ** *p* < 0.01; * *p* < 0.05; ns, not significant.

Since the Immunomodulatory mAbs show their most potent anti-tumor effects by activating tumor infiltrating lymphocytes against cancer cells, we also investigated the anti-tumor effects of the novel anti-PD-1, anti-PD-L1 or anti-CTLA-4 mAbs, used as single agents or in combination on tumor cells co-cultured with hPBMCs. To this aim, the BT-549 and MDA-MB-231 tumor cells, that were found to be the most sensitive to the previous treatments, were incubated in 96-well flat-bottom plates with hPBMCs at effector:target ratio of 5:1 for 48 h in the absence or presence of single agent treatments or their combination. This effector target ratio was chosen as it was previously successfully used to measure the effects of Ipilimumab and other immunomodulatory mAbs on in vitro co-cultures of tumor cells and lymphocytes [5,37,38]. In parallel assays, co-cultures of untreated or treated cells with an unrelated mAb (100 nM) were used as negative controls, and co-cultures of cells treated with Ipilimumab or Nivolumab, were tested as positive controls. After the treatments, the cell supernatants were collected and analyzed for Lactate Dehydrogenase (LDH) release as a marker of tumor cell lysis. As shown in Figure 4A and Figure 5A, the PD-1_1, PD-L1_1 and ID1 mAbs, when used as single agents (black and grey bars), showed stronger cytotoxic effects than the clinically validated Nivolumab and Ipilimumab, by inducing an increase of LDH release (~30%) from tumor cells. More interestingly, the cytolytic effects obtained with the combinations of ID1 with PD-1_1 or with PD-L1_1 mAb (striped bars) on both the indicated cell lines, were significantly higher than the single agent treatments (see Figure 4A and Figure 5A and Figure S1). These combinations seem to significantly improve not only the anti-tumor effects of each mAb, but they also show more potent anti-tumor effects with respect to the Nivolumab-Ipilimumab combinatorial treatment, especially on BT-549 cell line. Moreover, when we combined Nivolumab with the novel anti-CTLA-4 ID1 mAb, we observed an enhanced effect on LDH release raising up to 50 and 60%, respectively on MDA-MB-231 and BT-549 tumor cell lines (Figure 4A and Figure 5A), respectively, thus indicating the higher efficiency of this combination, compared to the lower effect (about 30%) observed when Nivolumab was combined with Ipilimumab.

In order to investigate whether the Immunomodulatory mAbs induce tumor cell lysis through the activation of hPBMCs, we examined the release of Interleukin 2 (IL-2) and Interferon gamma (IFN-γ). Thus, the supernatant, collected from the co-cultured cells treated as indicated above, was analyzed by ELISA for the detection of IL-2 and IFN-γ. As reported in Figure 4B and Figure 5B, all the mAbs used alone or in combination, increased the secretion of both cytokines, with respect to co-cultures of hPBMCs and untreated or treated tumor cells with an unrelated mAb (white and light grey bars), thus confirming their ability to stimulate lymphocytes improving immune responses against cancer. The highest release of IL-2 and IFN-γ was observed on both the cell lines with the combination of PD-L1_1 and ID1 (striped bars), at the concentration of 100 nM, which reached levels of IL-2 up to 6000 pg/mL and of IFN-γ up to 4500 pg/mL.

### 2.3. Effects of Combinatorial Treatments on Co-Cultures of hPBMCs and Cardiac Cells

**Figure 6:**
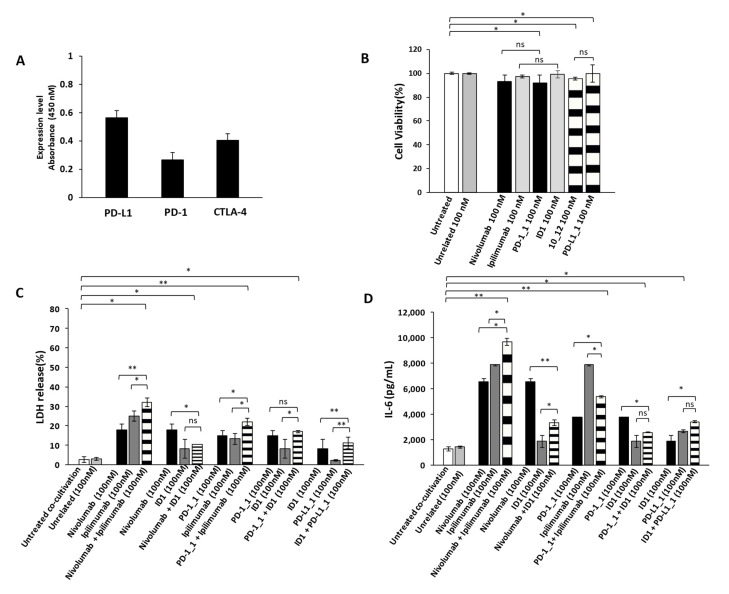
Cardiotoxic and pro-inflammatory effects induced by Immunomodulatory mAbs on human fetal cardiomyocytes. (**A**) Cell ELISA assay performed on HFC cardiac cells by using commercial anti-PD-1, anti-PD-L1 or anti-CTLA-4 mAbs (200 nM). (**B**) Cell viability assay on human fetal cardiomyocytes, treated for 48 h with anti-PD-1(black bars), anti-PD-L1 (striped bars) or anti-CTLA-4 (grey bars) mAbs. Cell viability is expressed as percentage of viable cells with respect to control untreated ones. Cells were counted by Trypan Blue exclusion test. (**C**) Cardiotoxic effects of immunomodulatory mAbs were analyzed by evaluating the LDH release on the supernatants of HFC cells co-cultured with hPBMCs, treated for 24 h with single mAbs (black and grey bars) or their combinations (striped bars). (**D**) Pro-inflammatory effects of immunomodulatory mAbs were analyzed by evaluating the secretion of IL-6 in the supernatant of the treated co-cultures. IL-6 is expressed as pg/mL. The values were reported as the mean of at least three determinations obtained in three independent experiments. Error bars depict means ± SD, ** *p* < 0.01; * *p* < 0.05; ns, not significant.

As previously reported in literature and also confirmed in this work, the combinatorial treatments of IC inhibitors increase the anti-tumor potency; however, they were found also responsible for a higher incidence of cardiotoxic events especially in patients treated with the combination of Nivolumab and Ipilimumab [37,38,39]. Thus, we decided to evaluate potential cardiotoxic side effects of the novel immunomodulatory mAbs and their combinations on Human Fetal Cardiomyocytes (HFC). We firstly evaluated the levels of expression of PD-1, PD-L1 and CTLA-4 on HFC and then we tested the effects of the mAbs on their growth (Figure 6A). As shown in Figure 6B, even though the ICs are expressed also on these cells, no significant effects were observed on HFC cell viability in the absence of immune cells.

We then investigated the effects of immunomodulatory mAbs and their combinations on co-cultures of HFC cells with hPBMCs, in order to compare their anti-tumor activity and eventual cardiotoxic side effects that could occur in vivo in cancer patients bearing solid tumors. To this aim, HFC cells were co-cultured with hPBMCs (effector:target ratio 5:1) and treated for 24 h with or without the indicated antibodies (100 nM). We used this concentration of mAbs, already used in previous studies [37] and chosen for the above-mentioned anti-tumor assays, to directly compare their effects on cardiac and tumor cells. At the end of incubation at 37 °C, the supernatant was collected and analyzed for LDH and IL-6 release, as a marker of inflammation of cardiac cells. As shown in Figure 6C, the combination of Nivolumab and Ipilimumab significantly induced the lysis of cardiac cells, whereas the corresponding combination of the novel PD-1_1 and ID1 mAbs (Figure 6C) showed a less marked effect, thus resulting in reduced toxicity for HFC cells (Figures S2A and S2B). Accordingly, the combination of Nivolumab and Ipilimumab shows the highest (Figure 6D) IL-6 pro-inflammatory cytokine secretion (~9700 pg/mL), thus confirming the cardiotoxic side effects of this combination. On the contrary, the combination of the other anti-CTLA-4 mAb ID1 with Nivolumab, PD-1_1 or PD-L1_1, even though equally or more effective on tumor cells than the combination of Ipilimumab and Nivolumab, showed much lower toxicity for HFC, as highlighted by reduced LDH release (Figure 6C) and lower levels of secreted pro-inflammatory IL-6.

## 3. Discussion

Immunotherapy completely revolutionized modern cancer therapy by enhancing anti-tumor effects of immune cells and overcoming some limits of conventional treatments. The understanding of mechanisms underlying the proliferation and regulation of T-cells paved the way to identify specific targets (the ICs), against which antibodies acting either as agonists for co-stimulatory receptors or antagonists for inhibitory receptors were generated, in order to improve immune responses of tumor infiltrating lymphocytes [3,4,9,11,40]. Recently, it has been reported the expression of ICs not only on immune cell populations [5,6,14], but also on cancer cells, where they seem to promote the evasion from anti-tumor immune responses [29].

Herein, we investigated the anti-tumor effects, on breast and lung cancer cells, of novel PD-1_1, PD-L1_1 and ID1 mAbs, previously generated in our laboratory [6,27,28,36] by an innovative phage display selection on activated lymphocytes. We tested them in comparison with the clinically validated Nivolumab and Ipilimumab. To this aim, we produced the anti-PD-1 mAb with IgG4 isotype, and anti-CTLA-4 mAb with IgG1 isotype, in order to better compare them to Nivolumab and Ipilimumab, IgG4 and IgG1, respectively, to evaluate their isotype-independent effects on tumor cells. We observed significant anti-tumor effects of all the anti-PD-L1, anti-PD-1 and anti-CTLA-4 mAbs on tumor cells even in the absence of T-cells, thus supporting the hypothesis that ICs could play an additional role in promoting tumor cell survival and proliferation [6,28]. Previous investigations of our group highlighted the ability of Ipilimumab, ID-1, PDL1_1 and 10_12 mAbs to affect MAPK pathway [6,28]. In particular, PD-L1_1 and 10_12 mAbs were found to inhibit the phosphorylation levels of Erk, P38 and JNK in SKBR-3 and CT-26 tumor cells, whereas Ipilimumab and ID-1 increased the phosphorylation of Erk in NK cells. Here, we show that all the anti-PD-1 and anti-PD-L1 mAbs are able to reduce Erk phosphorylation and, unexpectedly, to induce a very significant increase of PD-1 and PD-L1 expression on lung and breast tumor cells. This observation has been strengthened by studies with agonistic PD-L1 and PD-1 recombinant proteins that showed opposite effects on tumor cells by leading to a significant reduction of PD-1 receptor and its PD-L1 ligand. The effects of antagonistic treatments leading to PD-1 and PD-L1 increased expression confirm the regulation of this receptor/ligand to be a key element, associated with a marked hyperphosphorylation of the NF-kB transcription factor. As previously reported in the literature, NF-kB phosphorylation could fasten its translocation into the nucleus, where it could likely induce PD-L1 and PD-1 transcription, by binding to the promoter of PD-L1 gene [8] or to the Conserved Region-C located upstream of PD-1 gene, as it occurs for PD-1 gene in macrophages [41], or it could induce the expression of CSN5, which is responsible for post-translational stabilization of PD-L1. Hence, we could assume that NF-kB regulates the expression of both PD-L1 [8] and its receptor, by increasing PD-1 and PD-L1 transcription, in case of treatments with antagonistic mAbs Nivolumab or 10_12. Furthermore, in parallel to the increased expression of PD-1 and PD-L1, a high expression of CTLA-4 was observed on tumor cells treated with PD-1/PD-L1 antagonists, whereas a decreased expression was detected in case of PD-1/PD-L1 agonistic treatments. These intriguing findings support the hypothesis of reciprocal modulation among these specific immune checkpoints and highlight for the first time the existence of a crosstalk also in tumor cells involving not only PD-L1 and its receptor PD-1, but also CTLA-4.

Thus, we show here how immunomodulatory mAbs can be used also as analytical and biotechnological tools to better clarify the role of immune checkpoints on a molecular level and to understand how they can affect their own and other receptors expression or downstream pathways involved in tumor cell proliferation and survival.

Since the immune checkpoint inhibitors (ICIs), such as immunomodulatory antibodies, have shown efficacy and have been approved for the treatment of several tumors [42,43,44,45,46,47,48,49], but their beneficial effects are still limited to 20–30% of the population, ongoing clinical trials in cancer patients include combinations of immunomodulatory mAbs specific for different targets to achieve additive or synergistic effects, in order to improve T cell anti-cancer activity. Indeed, a dramatic increase of efficacy of Nivolumab and Ipilimumab combinatorial treatment with respect to monotherapies was observed in melanoma patients [39].

However, inhibition by mAbs of the T cell co-inhibitory pathways or activation of the co-stimulatory pathways might generate serious risk of cardiovascular events such as, myocarditis and pericarditis, even though in a low percentage of treated patients [39,50,51,52,53,54,55,56,57,58], that should be taken into consideration particularly in combinatorial therapeutic approaches.

Thus, we investigated whether it was possible to identify the most efficient combinations of immunomodulatory mAbs for achieving potent anti-tumor efficacy associated with the lowest adverse side effects by using novel simple and predictive in vitro models based on co-cultures of tumor cells or human fetal cardiomyocytes with hPBMCs.

As a proof of concept, we firstly tested the treatment of Nivolumab and Ipilimumab, previously associated with higher anti-tumor efficacy but also to cardiac injures in cancer patients [39]. We found that this combination induced a significant release of LDH (more than 30%) in co-cultures of cardiomyocytes and lymphocytes associated with significant secretion of pro-inflammatory IL-6, which has been previously reported to be involved in the etiopathogenesis of myocarditis [37,38,59,60].

On the other side, combinations including the novel mAbs, such as PD-1_1 or PD-L1_1 with ID1 [27,28], not only showed a more potent cancer cell killing activity in co-cultures of tumor cells and hPBMCs, inducing about 50% and 40% of cells lysis, respectively, associated with secretion of higher levels of IL-2 and IFN-γ, but proved also to be lower cardiotoxic than the Nivolumab–Ipilimumab combination in co-cultures of cardiomyocytes and lymphocytes showing reduced cell lysis and lower levels of IL-6 pro-inflammatory cytokine. In particular the most interesting combination including ID1 and PD-L1_1 shows the highest anti-tumor cytotoxicity with the lowest cardiotoxicity.

Thus, we conclude that these co-cultures system-based assays, proposed here, could become very useful in the future to test also many other combinatorial treatments of emerging immunomodulatory mAbs against different ICs (such as those specific for Lag-3, TIM-3, ICOS and others) to early predict not only their anti-tumor efficacy but also their side adverse events, thus allowing for the early screening of most potent and safe combinatorial therapeutic regimens.

## 4. Materials and Methods

### 4.1. Antibodies and Human Recombinant Proteins

**Figure 7:**
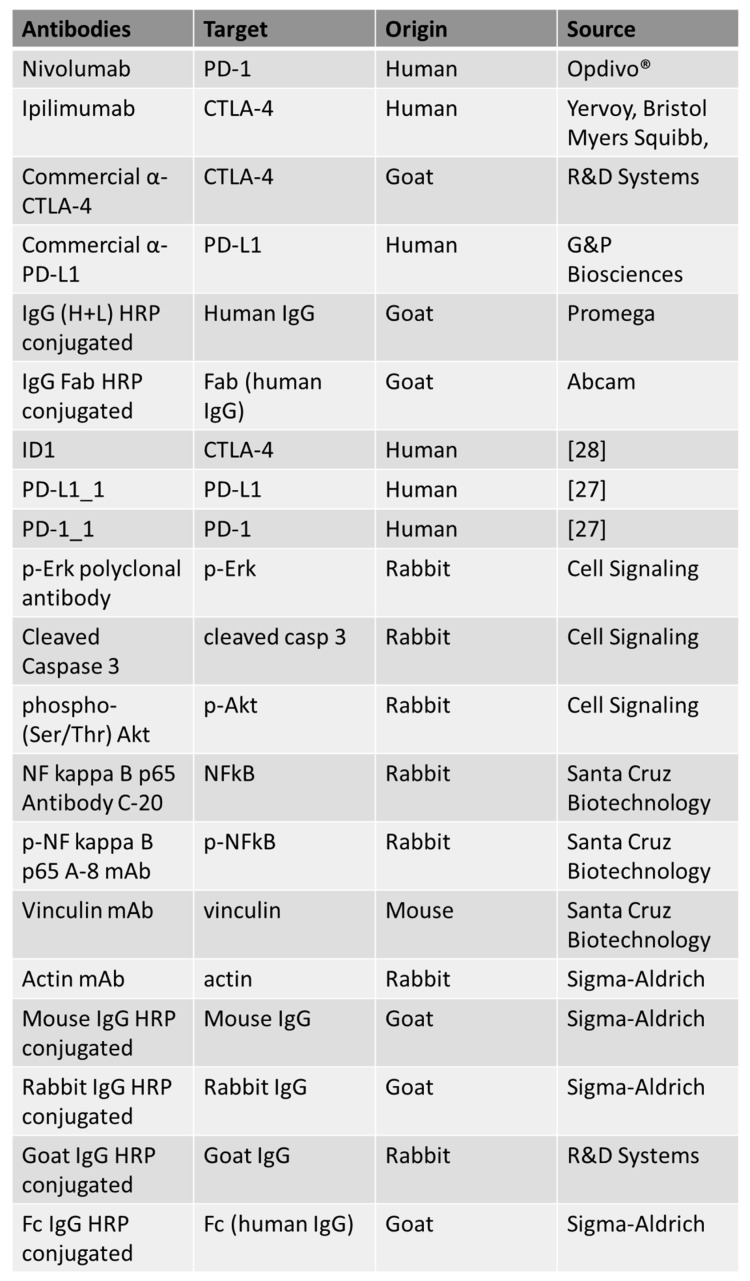
List of the antibodies used in this work. For each antibody used, the specific target, origin and source are indicated.

The following antibodies were used, as reported in Figure 7: anti-human PD-1 human mAb Nivolumab (Opdivo^®^); anti-CTLA-4 mAb Ipilimumab (Yervoy, Bristol Myers Squibb, NY, USA); anti-human PD-L1 human mAb (G&P Biosciences, Santa Clara, CA, USA); commercial Human CTLA-4 Antibody (R&D Systems, Minneapolis, MN, USA); anti-human IgG (H+L) HRP conjugate antibody (Promega, Madison, WI, USA); HRP-conjugated anti-human IgG (Fab’)2 goat monoclonal antibody (Abcam, Cambridge, UK); anti-human p-Erk rabbit polyclonal antibody, anti-human Cleaved Caspase-3 rabbit polyclonal antibody and anti-phospho-(Ser/Thr) Akt (all from Cell Signaling, Danvers, MA, USA); NF kappa B p65 Antibody C-20 polyclonal antibody, p-NF kappa B p65 Antibody A-8 monoclonal antibody and anti-vinculin monoclonal antibody (all from Santa Cruz Biotechnology, Inc. Dallas, TX, USA); anti-actin antibody (Sigma-Aldrich, Darmdstadt, Germany); anti-goat IgG HRP-conjugated (R&D Systems, Minneapolis, MN, USA); anti-human IgG (Fc-specific) HRP-conjugated, anti-Mouse IgG HRP conjugate and anti-rabbit IgG HRP conjugate (all from Sigma). The following recombinant proteins were used: human PD-1/Fc and human PD-L1/Fc (all from R&D Systems). ID1 (anti-CTLA-4), PD-1_1 (anti-PD-1), PD-L1_1 and 10–12 (anti-PD-L1) monoclonals were produced and purified as previously described [61], taking advantage from the enhanced cell line HEK293_ES1 expressing a long non-coding SINEUP RNA [62].

### 4.2. Cell Cultures

MDA-MB-231 breast cancer cells were cultured in Dulbecco’s Modified Eagle’s Medium (DMEM, Gibco, Life Technologies, Paisley, UK). BT-549 breast cancer cells were cultured in Roswell Park Memorial Institute 1640 Medium (RPMI 1640, Gibco, Life Technologies, Paisley, UK). A-549 lung cancer cells were cultured in Kaign’s Modification of Ham’s F-12 Medium (F-12K, American Type Culture Collection, Manassas, VA, USA). Human fetal cardiomyocytes were cultured in Cardiac Myocyte Medium (CMM, Innoprot, Derio—Bizkaia, Spain), according to the manufacturer’s recommendations.

Cell lines were purchased from the American Type Culture Collection (ATCC) and cultured in humidified atmosphere containing 5% CO_2_ at 37 °C. The media were supplemented with 10% (vol/vol) heat-inactivated fetal bovine serum (FBS, Sigma, St Louis, MO, USA) and were used after addition of 50 U/mL penicillin, 50 μg/mL streptomycin, 2 nM L-glutamine (all from Gibco, Life Technologies, Paisley, UK).

### 4.3. Enzyme-Linked Immunosorbent Assays (ELISA)

To check the expression level of PD-1, PD-L1 and CTLA-4 cell ELISA assays were performed on cancer cells and human fetal cardiomyocytes. Cells were plated in triplicates into a NuncTM round-bottom 96-well plate at the density of 2 × 10^5^ cells/well and incubated with a blocking solution (PBS/BSA 6%) for 20 min at RT. Then, cells were incubated in the absence or in the presence of Nivolumab, anti-PD-L1(G&P Biosciences) or anti-CTLA-4(R&D Systems) mAbs at a concentration of 200 nM in PBS/BSA 3% buffer solution for 2 h at RT with gentle agitation. After the incubation with the primary antibodies, the plates were washed with PBS and incubated with an appropriate HRP-conjugated antibody for 1 h at room temperature. After extensive washes, 3,3′,5,5′-Tetramethylbenzidine (TMB) (Sigma-Aldrich, St. Louise, MO, USA) reagent was added for 10 min before quenching with an equal volume of 1 N HCl. Absorbance at 450 nm was measured by the Envision plate reader (Perkin Elmer, 2102, San Diego, CA, USA).

### 4.4. Western Blotting Analyses

MDA-MB-231, BT-549 cells and A-549 cells were plated at a density of 6 x 10^5^ cells/well and incubated for 72 h at 37 °C in the absence or in presence of Nivolumab, Ipilimumab or 10_12 mAbs, at a concentration of 200 nM, or with PD-1/Fc or PD-L1/Fc chimeric proteins at a concentration of 1 μg/mL. Cells were scraped and centrifuged at 1200 rpm for 5 min; the cell pellets were lysed in a buffer containing 10 mM Tris-HCl (pH 7.4), 0.5% Nonidet-P-40, 150 mM NaCl and 1 mM Sodium orthovanadate (Sigma-Aldrich, St. Louise, USA), in the presence of protease inhibitors (Roche, Indianapolis, USA). After incubation on ice for 20 min, the extracts were clarified by centrifugation at 12,000 rpm for 15 min at 4 °C. Protein concentration was determined by the Bradford colorimetric assay (Sigma-Aldrich, USA) and Western blotting analyses were performed by incubating the membranes with the following primary antibodies: α-PD-1 Nivolumab (Opdivo^®^), commercial human anti-PD-L1 Ab (G&P Biosciences), commercial human CTLA-4 Antibody (R&D Systems, Minneapolis, MN, USA), anti-human p-Erk rabbit polyclonal antibody; anti-human Cleaved Caspase-3 rabbit polyclonal antibody; anti-phospho-(Ser/Thr) Akt (all from Cell Signaling, Danvers, MA, USA), NF kappa B p65 Antibody C-20 polyclonal antibody or p-NF kappa B p65 Antibody A-8 monoclonal antibody (from Santa Cruz Biotechnology), followed by the HRP-conjugated secondary antibody. Specifically, goat anti-human polyclonal IgG (Fc-specific) was used for the detection of α-PD-1 and α-PD-L1 primary antibodies; rabbit anti-goat polyclonal IgG HRP-conjugated (R&D Systems, Minneapolis, MN, USA) for the detection of α-CTLA-4 antibody, and goat anti-rabbit polyclonal IgG HRP-conjugated for all the other antibodies. To normalize the intensity of the bands the membranes were incubated with anti-actin or anti-vinculin mAbs (respectively from Sigma-Aldrich and Santa Cruz Biotechnology), followed by goat anti-rabbit polyclonal IgG HRP-conjugated or goat anti-Mouse polyclonal IgG HRP conjugated secondary antibodies (both from Sigma).

### 4.5. Cell Growth Inhibition Assays

For the evaluation of the effects induced by mAbs, cancer cells were plated at a density of 5 × 10^3^ cells/well, whereas HFC were plated at a density of 1 × 10^4^ cells/well in 96-well flat-bottom plates for 16 h. Then, they were incubated in the absence or in presence of Nivolumab, Ipilimumab, novel mAbs (PD-1_1, 10–12, PD-L1_1, ID1) or an unrelated IgG control at the concentrations of 100 nM, for 72 or 48 h, respectively. Viable cells were counted by the trypan blue exclusion test and cell survival was expressed as percent of viable cells with respect to the untreated cells used as negative control.

### 4.6. Cytotoxicity Assays and LDH Detection

Co-cultures of cells with hPBMCs (effector: target ratio 5:1) were treated to test the cytotoxic effects of mAbs used as single agents or in combination. Tumor cells were plated in 96-well flat-bottom plates at the density of 1 × 10^4^ cells/well, whereas HFC cardiac cells were plated at a density of 1.5 × 10^4^, for 16 h. Then, hPBMCs from healthy donors were added in the absence or presence of Nivolumab, Ipilimumab, PD-1_1, PD-L1_1 or ID1, used alone or in combination at a concentration of 100 nM, at 37 °C for 48 or 24 h. Untreated cells and cells incubated with an unrelated IgG control (100 nM) were used as negative controls. Tumor and cardiac cell lysis were evaluated by measuring the release of lactate dehydrogenase (LDH) in the supernatant of co-cultures described above by LDH detection kit (Thermofisher Scientific, Rockford, IL, USA), following the manufacturer’s recommendations. Cell lysis was analyzed by measuring the fold increase of LDH in the presence of each treatment, with respect to the amount present in the supernatant of co-cultures untreated or treated with an unrelated mAb. Cytolysis values were obtained from at least three independent values.

### 4.7. Cytokine Secretion Assays

The secretion of Interleukin 6, Interleukin-2 and IFN-γ in co-cultures of cells with hPBMCs were evaluated by ELISA assays. Briefly, after treatments culture supernatants were centrifuged and treated for quantification of human IL-6 (ELISA MAX^TM^ Deluxe Set Human IL-6, BioLegend, San Diego, CA, USA), of IL-2 and IFN-γ (DuoSet ELISA, R&D Systems, Minneapolis, MN, USA), according to the producer’s recommendations. Concentration values were reported as the mean of at least three determinations.

### 4.8. Statistical Analyses

Error bars were calculated on the basis of the results obtained by at least three independent experiments. Statistical analyses were assessed by Student’s t-test (two variables). Statistical significance was established as *** *p* ≤ 0.001; ** *p* < 0.01; * *p* < 0.05.

## 5. Conclusions

We investigated here for the first time on the cis-interaction between some Immune Checkpoints in tumor cells by using novel human anti-PD-1, anti-CTLA-4 and anti-PD-L1 mAbs. Additionally, we set up an in vitro system to early predict the efficacy and eventual cardiotoxic side effects of combinatorial treatments of immunomodulatory mAbs useful to test in the future also other combinatorial treatments of emerging immunomodulatory mAbs against different ICs, thus allowing for the early screening of most potent and safe combinatorial therapeutic regimens.

## Supplementary Materials

The following are available online at https://www.mdpi.com/article/10.3390/cancers13122858/s1, Figure S1. Anti-tumor effects of novel immunomodulatory mAbs tested in parallel with Nivolumab or Ipilimumab, Figure S2. Cardiotoxic effects of novel immunomodulatory mAbs tested in parallel with Nivolumab or Ipilimumab, Figure S3. Full length blots of Figure 1A, Figure S4. Full length blots of Figure 2B, Figure S5. Full length blots of Figure 3A.
