# Supplementary material for: Immunomodulatory mAbs as Tools to Investigate on Cis-Interaction of PD-1/PD-L1 on Tumor Cells and to Set Up Methods for Early Screening of Safe and Potent Combinatorial Treatments
Source: Cancers (Basel). 2021 Jun 8;13(12):2858. doi: 10.3390/cancers13122858 (PMC8230074; doi:10.3390/cancers13122858)
Supplement: Supplementary file 1 [file cancers-13-02858-s001.zip › cancers-1217297-supplementary.pdf]

cancers

Supplementary Materials: Immunomodulatory mAbs as Tools to Investigate on Cis-Interaction of PD-1/PD-L1 on Tumor Cells and to Set Up Methods for Early Screening of Safe and Potent Combinatorial Treatments

Cinzia Vetrei, Margherita Passariello, Guendalina Froechlich, Rosa Rapuano Lembo, Nicola Zambrano and Claudia De Lorenzo

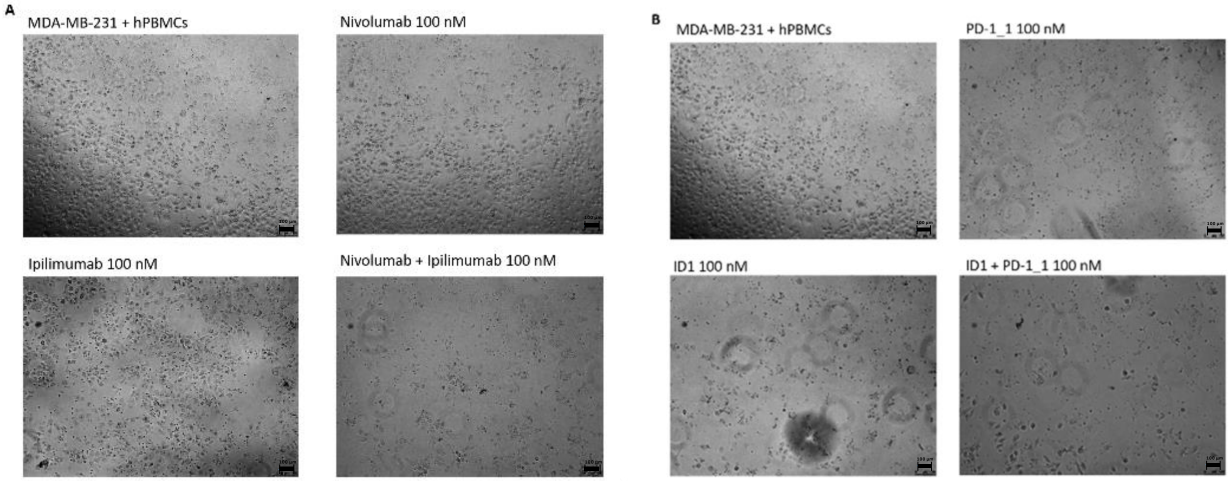

Figure S1. Anti-tumor effects of novel immunomodulatory mAbs tested in parallel with Nivolumab or Ipilimumab. Representative images of MDA-MB-231 tumor cells treated for 48 h with Nivolumab, Ipilimumab and their combination (**A**) or the novel anti-PD-1, anti-CTLA-4 mAbs and their combination (**B**) tested in the presence of lymphocytes. Magnification 0,63×, 10.0× digital zoom. Scale bar = 100 µm.

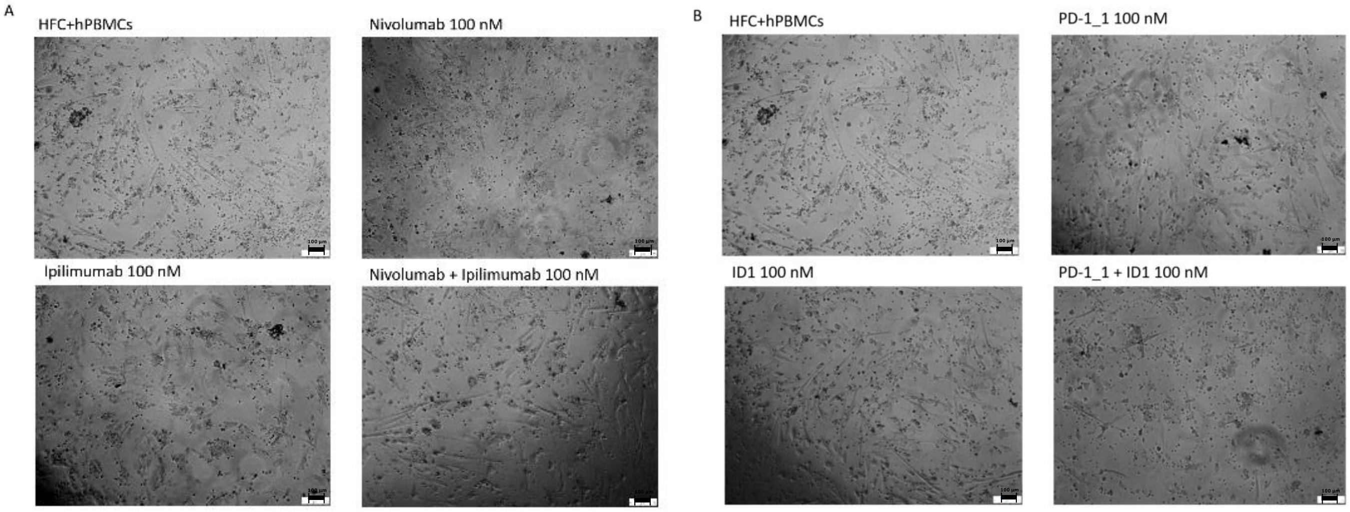

Figure S2. Cardiotoxic effects of novel immunomodulatory mAbs tested in parallel with Nivolumab or Ipilimumab. Representative images of HFC treated for 24 h with Nivolumab, Ipilimumab and their combination (**A**) or the novel anti-PD-1, anti-CTLA-4 mAbs and their combination (**B**) tested in the presence of lymphocytes. Magnification 0,63×, 10.0× digital zoom. Scale bar = 100 µm.

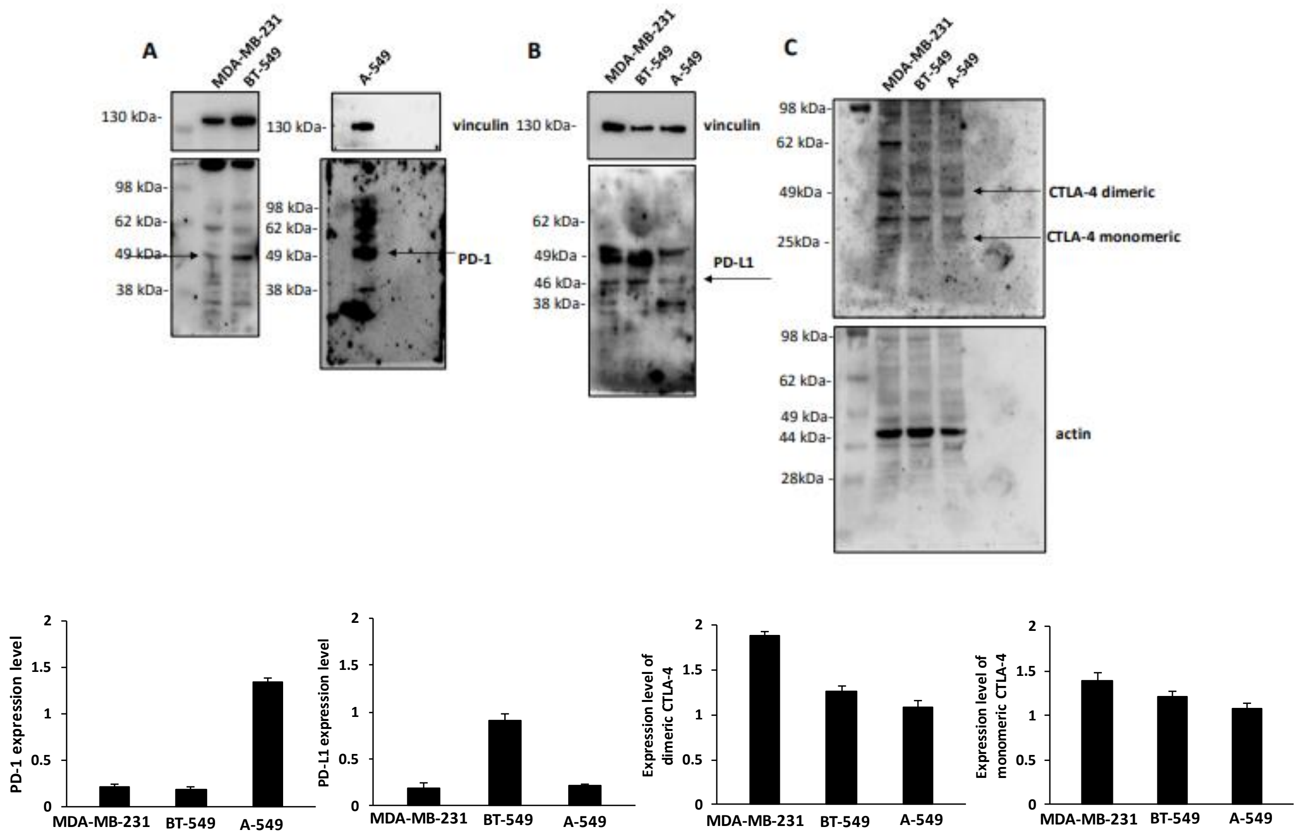

Figure S3. Full length blots of Figure 1A. (**A**) Western blotting for the detection of PD-1 levels: the upper parts of the filters were stained with an anti-Vinculin mAb; the lower parts of the filters were stained with the anti-PD-1 human mAb. (**B**) Western blotting for the detection of PD-L1 levels: the upper part of the filter was stained with an anti-Vinculin mAb; the lower part of the filter was stained with the anti-PD-L1 human mAb. (**C**) Western blotting for the detection of CTLA-4 levels: the full lenght filter was firstly incubated with anti-CTLA-4 human polyclonal antibody and then with an anti-Actin mAb. The intensity of the bands corresponding to ICs was normalized to actin or vinculin and their ratio is reported in the graphics as protein expression levels.

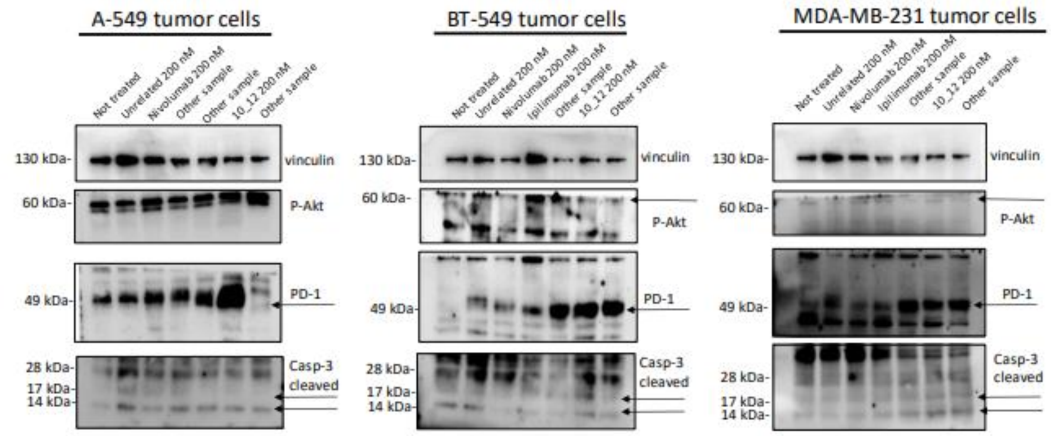

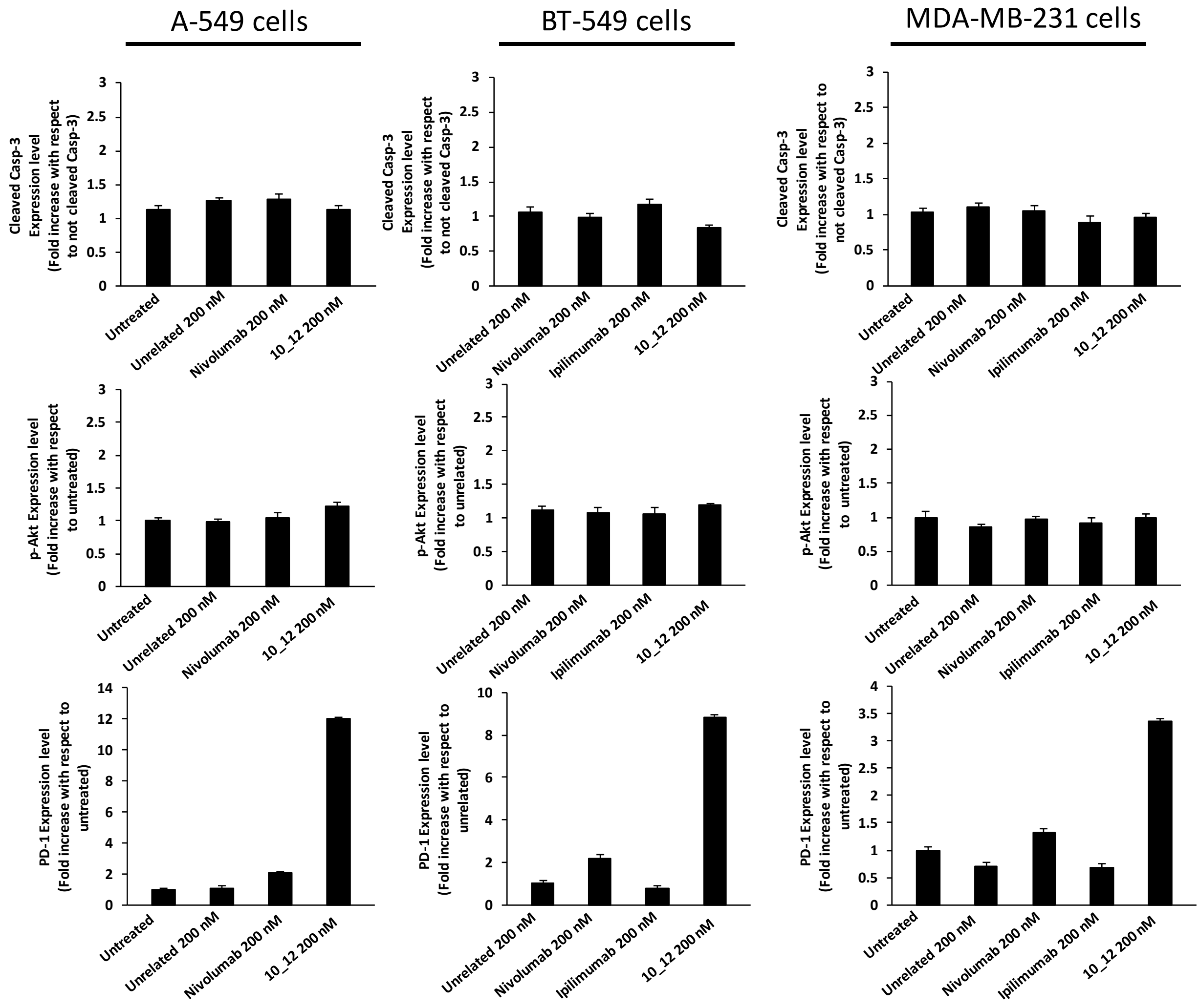

Figure S4. Full length blots of Figure 2B. Western blotting analyses of extracts from A-549, BT-549 and MDA-MB-231 tumor cells treated with Nivolumab, Ipilimumab or 10_12 mAbs for 72 h. The extracts from the three cell lines were loaded on three filters (as indicated), than the different portions of each filter (cut on the basis of reference MW) were stained with anti-p-Akt polyclonal antibody, anti-Caspase 3 cleaved polyclonal antibody, anti-PD-1 or anti-Vinculin mAbs, as indicated by the arrows. The intensity of the bands corresponding to the detected proteins was normalized to vinculin; protein levels are expressed as fold increase with respect to cells untreated or treated with an unrelated IgG; cleaved Caspase-3 level is expressed as fold increase with respect to uncleaved Caspase-3.

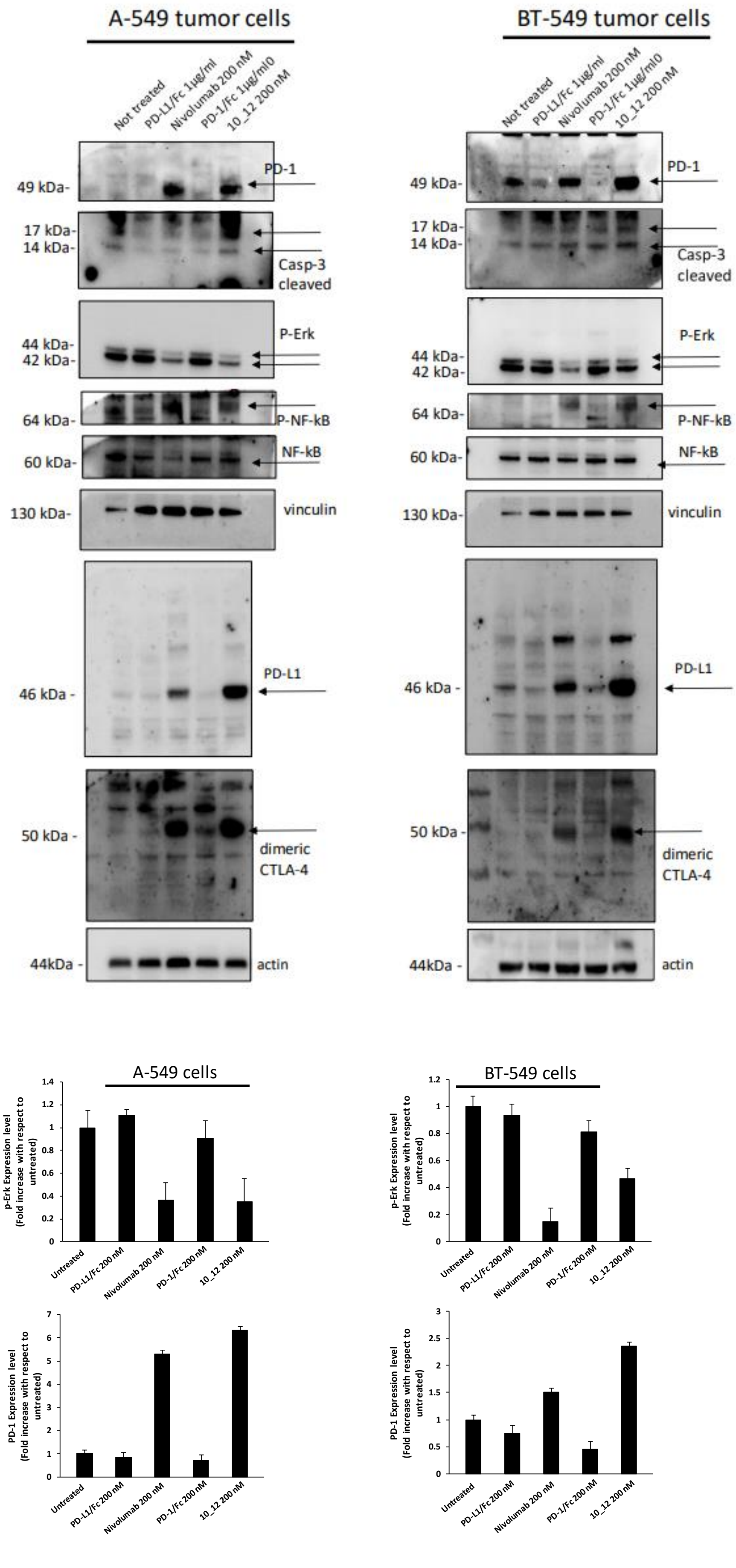
A-549 tumor cells
BT-549 tumor cells
Not treated
PD-L1/Fc 1µg/ml
Nivolumab 200 nM
PD-1/Fc 1µg/ml0
10_12 200 nM
49 kDa-
PD-1
17 kDa-
14 kDa-
Casp-3 cleaved
44 kDa-
42 kDa-
P-Erk
64 kDa-
P-NF-kB
60 kDa-
NF-kB
130 kDa-
vinculin
46 kDa -
PD-L1
50 kDa -
dimeric CTLA-4
44kDa -
actin
A-549 cells
BT-549 cells
p-Erk Expression level (Fold increase with respect to untreated)
PD-1 Expression level (Fold increase with respect to untreated)
Untreated
PD-L1/Fc 200 nM
Nivolumab 200 nM
PD-1/Fc 200 nM
10_12 200 nM

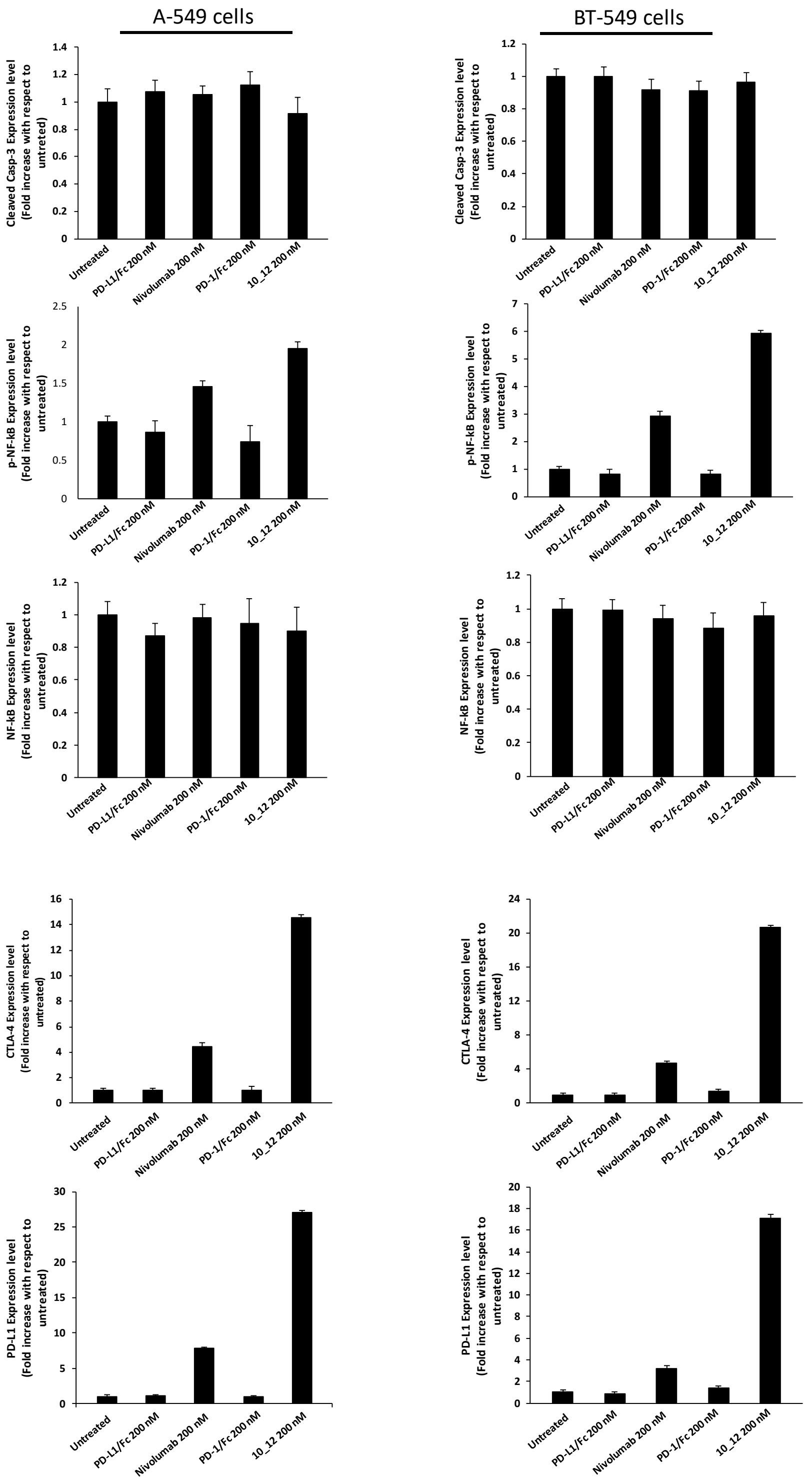

Figure S5. Full length blots of Figure 3A. Western blotting analyses of extracts from A-549 and BT-549 tumor cells treated as indicated for 72 h. The extracts from the two cell lines were loaded in duplicates on two filters (as indicated), than the different portions of each filter (cut on the basis of reference MW) were stained with anti-p-Erk polyclonal antibody, anti-Caspase 3 cleaved polyclonal antibody, anti-PD-1 mAb, anti-NF-kB polyclonal antibody, anti-p-NF-kB, anti-Vinculin mAbs or with anti-CTLA-4 polyclonal antibody, anti- PD-L1 and anti-Actin mAbs. The intensity of the bands corresponding to the detected proteins was normalized to vinculin or actin; protein levels are expressed as fold increase with respect to untreated cells.
